# Analytically grounded full-wave methods for advances in computational electromagnetics

**DOI:** 10.1098/rsta.2024.0356

**Published:** 2025-08-14

**Authors:** Mario Lucido, Kazuya Kobayashi, Alexander I. Nosich, Carlos Pérez Arancibia, Ana Vukovic

**Affiliations:** ^1^ Department of Electrical and Information Engineering “Maurizio Scarano”, University of Cassino and Southern Lazio, Cassino, Italy; ^2^ EUt+ Institute of Nanomaterials and Nanotechnologies-EUTINN, European University of Technology, European Union; ^3^ Department of Electrical, Electronic, and Communication Engineering, Chuo University, Hachioji, Tokyo, Japan; ^4^ Laboratory of Micro and Nano Optics, O Ya Usikov Institute for Radiophysics and Electronics National Academy of Sciences of Ukraine, Kharkiv, Ukraine; ^5^ Mathematics of Computational Science group, University of Twente, Enschede, Overijssel, The Netherlands; ^6^ George Green Institute for Electromagnetics Research, University of Nottingham, Nottingham, UK

**Keywords:** computational electromagnetics, integral equations, wave scattering

## Abstract

This Theme Issue is a collection of original research and review papers focused on developing a class of well-established and innovative analytically grounded full-wave methods and their applications in computational electromagnetics. These methods are notable for their guaranteed convergence, meaning that the approximate solution obtained by discretizing and truncating the equation governing the problem at hand tends to the exact solution if the truncation order gets larger. Hence, unlike the numerical approximations with no mathematically guaranteed convergence, they do not require post-validation. Moreover, highly accurate solutions are reconstructed with a low computational cost, thus allowing a real-time, precise and exhaustive parametric analysis of various critical structures and complicated physical phenomena. To conclude, the obtained solutions deliver trusted physical results within a reasonable time and without false effects and, therefore, can serve as a reference for validating general-purpose commercial software.

This article is part of the theme issue ‘Analytically grounded full-wave methods for advances in computational electromagnetics’.

## 1. Introduction

The push towards innovations in nano-optics and photonics, and the use of cutting-edge materials like graphene—alongside the drive to extend wireless technologies into millimetre-wave and THz frequency bands—has spurred the development of versatile, full-wave electromagnetic simulation tools. Among the various approaches, integral equation methods stand out due to two key advantages: they inherently enforce the correct behaviour of the electromagnetic fields at infinity (radiation condition) and operate with unknowns confined to bounded regions, simplifying computations [1].

However, when a problem cannot be naturally cast as a Fredholm integral equation of the second kind, or when its discretization fails to preserve that structure, or if discretization does not lead to the matrix equations with diagonal predominance, the accuracy and reliability of the solution become uncertain [2]. This is because of the absence of convergence, understood as the possibility of minimizing computation error, which is a certain norm, all the way to machine precision of the computer used, by increasing the discretization order, i.e. the final matrix truncation number.

Although rarely recognized in the engineering community, such an unfortunate situation happens in a broad class of wave propagation, radiation and scattering problems, involving open scatterers, objects with wedges, planar surfaces, etc., that can be equivalently formulated as singular integral equations, for which the Fredholm theory cannot be applied. In such cases, ex-post validation, i.e. comparisons with analytical solutions, experimental measurements or asymptotic results, is crucial, since there is no general assurance of the true solution’s existence and no measure of the nearness, in any sense, of the numerical approximation to the true solution. As a result, there is no instrument to improve the accuracy of the obtained approximations systematically. This is a common limitation of general-purpose commercial software. Moreover, these tools often struggle with the analysis of advanced structures involving ultrathin materials (e.g. graphene sheets), infinite or open boundaries, or with capturing complex phenomena like natural mode resonances in the THz and optical ranges, making such problems challenging or even unmanageable for standard software.

On the other hand, all these troubles can be completely overcome using analytically grounded full-wave methods, i.e. methods for which the convergence of the discretization scheme adopted is guaranteed. Among them, the Method of Analytical Regularization (MAR)—including, among others, the Modified Wiener–Hopf technique, the Abel Transform technique and the Riemann–Hilbert Problem technique—the Method of Analytical Preconditioning (MAP), also known as Regularizing Galerkin technique, and various forms of the Nyström Discretization technique deserve to be mentioned.

MAR has been a cornerstone in addressing challenging integral equations for decades [3]. MAR encompasses techniques aimed at transforming various strongly singular integral equations and weakly singular first-kind integral equations into the integral or matrix equations of the Fredholm second kind, ensuring well-posedness and guaranteed convergence. The underlying idea is conceptually straightforward yet mathematically elegant: isolate a suitably chosen most singular part of the integral operator and invert it analytically. This reference operator can be tailored to the specific problem—it is often taken as the static, high-frequency or canonical geometry component of the original operator. To carry out its inversion, powerful tools from functional analysis are employed, such as the Titchmarsh theorem, Wiener–Hopf factorization, Carleman formula, Cauchy and Abel transforms and Sokhotski–Plemelj theorem in the Riemann–Hilbert Problem theory. Moreover, for problems involving canonical shapes, separation of variables is frequently applied to derive explicit solutions to the invertible reference equations.

In a broad class of problems, analytical regularization and discretization of an integral equation are carried out simultaneously, giving rise to what is known as MAP [4]. This technique involves selecting the eigenfunctions of a carefully chosen singular part of the integral operator—typically a suitable part including the most singular one—as the expansion functions. In such a case, the Galerkin projection works as a perfect preconditioner, resulting in a Fredholm second-kind matrix equation. More generally, Fredholm theory remains applicable if the discretized operator can be written as the sum of an invertible operator (with a continuous inverse on both sides) and a compact (completely continuous) operator. When the convergence is guaranteed, numerical accuracy can be easily controlled by the matrix truncation order. In principle, the error can be brought to machine precision, which is unthinkable for today’s popular commercial codes.

The Wiener–Hopf technique [5] provides a rigorous analytical framework that yields exact solutions for wave diffraction and scattering from the perfectly electrically conducting (PEC) half-plane. Therefore, it can be used to build MAR-type solutions for certain more complicated geometries, ‘modified’ with respect to the halfplane—e.g. a finite PEC strip or several PEC halfplanes. It explicitly incorporates all the mathematical requirements, including the boundary, radiation and edge conditions, into the analysis and has been applied to various areas in science and engineering, such as electromagnetics, acoustics, elastic wave propagation and fluid dynamics. Moreover, in recent years, the range of applications of the Wiener–Hopf technique has been systematically extended to model complex mathematical-physics wave-scattering problems in various frontier disciplines, including nano-photonics, biomechanics and metamaterials.

The Nyström Discretization technique [6] allows for converting integral equations, particularly those encountered in electromagnetics, into a system of linear equations using the theorems of the quadrature approximations of singular integrals. Depending on the problem at hand, a suitable choice of the quadrature rule leads to guaranteed convergence, that, in the context of the Nyström method, refers to the assurance that as the number of quadrature points used in the numerical integration is increased, that is, the discretization refined, the approximate solution converges to the exact solution of the singular integral equation. According to the corresponding approximation theorems, this is provided by the diagonal predominance in the matrix equations obtained.

## 2. Theme Issue articles overview

This Theme Issue aims to collect the more recent developments in the analytically grounded full-wave methods and their applications for the new challenging frontiers of computational electromagnetics. In this section, a brief description of the contents of the articles in this issue will be presented.

In the article by Herasymova *et al*. [7], the infrared-range diffraction radiation from finite configurations of circular graphene-covered dielectric nanowires excited by the density-modulated beam of charged particles is addressed using the MAR. Here, the problem is transformed into a well-conditioned algebraic equation for the field expansion coefficients using the separation of variables and the addition theorem for the cylindrical functions, thus leading to the explicit inversion of the single-wire part of the problem. Kuryliak & Lysechko [8] apply a MAR technique based on the Abel integral transformation to study the scalar wave diffraction from an open-ended sphere-conical cavity, which is of significant interest in applied physics and engineering to model waveguide probes in microwave diagnostics, tripod-supported concave spherical reflectors and antennas, surface defects, etc. In the article by Oğuzer [9], the focusing ability of an electrically large thin dielectric parabolic reflector sandwiched between graphene covers illuminated by an E-polarized plane wave is addressed using the MAR based on a Fourier inversion procedure and the Riemann–Hilbert Problem technique. In their review article, Vinogradova & Smith [10] show the application of MAR based on the Abel integral transform to the electromagnetic wave scattering from perfectly electrically conducting (PEC) arbitrary slotted cylinders and axisymmetric thin-walled shells with one or two apertures.

The still not thoroughly addressed problem of handling entire unknowns and exponential phase factors in the solution of Wiener–Hopf equations, which is present each time the spectral-domain Wiener–Hopf method is applied to scattering problems involving finite penetrable/impenetrable regions, e.g. strips, slots, slits, bricks and cylindrical structures of various shapes that can be staggered concerning each other, is successfully addressed by Daniele & Lombardi [11]. Moreover, a review paper on the application of the Wiener–Hopf technique to the rigorous analysis of the radar cross section of two-dimensional cavities formed by a terminated, semi-infinite/finite parallel-plate waveguide with three-layer material loading is proposed by He *et al*. [12].

The transmission of the electromagnetic field through a circular aperture in the PEC plate is evaluated in the paper by Lovat *et al*. [13]. The problem, formulated as a set of dual integral equations for the equivalent magnetic source on the hole, is regularized by means of MAP, thus leading to a fast convergence. A variant of MAP, specifically called the Helmholtz–Galerkin technique, is adopted by Lucido [14] to efficiently regularize a suitable system of spectral-domain integral equations, resulting in the plane-wave scattering from a finite set of coplanar thin circular resistive discs in free space. Schettino *et al*. [15] regularize the electric field integral equations, formulating the plane-wave scattering from a finite-length closed PEC circular cylinder using MAP with expansion functions, which are the eigenfunctions of the singular part of the integral operator, reconstructing the field behaviour on the wedges. Zinenko *et al*. [16] use MAP with the weighted Chebyshev polynomials to accurately establish the threshold conditions for the natural modes of the microsize plasmonic laser shaped as an infinite flat graphene strip grating symmetrically embedded into the gain-material layer. It is worth noting that the canonical-shape MAR technique used in [7] can also be considered as a MAP scheme with the trigonometric polynomials, in the local coordinates of each circular wire, as expansion functions.

Dushkin [17] develops a variant of the Nyström scheme for the convergent numerical solution of the Cauchy-singular integral equations devised in analysing the scattering from non-PEC strips and strip gratings with and without a screen. Kaliberda & Pogarsky [18] use the mathematically grounded method of hyper-singular integral equations and meshless Nyström-type algorithm to study the modification of the radar cross section of a circular dielectric cylinder with coplanar graphene strips inside, focusing on the variation of the chemical potential and the excitation of associated plasmon resonances. Li & Lu [19] extend the perfectly matched layer-boundary integral equation method, combined with a suitable discretization technique of the Nyström type, to a two-dimensional electromagnetic scattering problem in a two-layer medium of a step-like interface. In the paper by Petropoulos & Turc [20], the scattering involving semi-infinite, infinite but not necessarily periodic, as well as large finite arrays of identical obstacles in free space in two dimensions, is studied using the decomposition numerical approach together with boundary integral equations and Nyström discretization. Tsalamengas [21], in the analysis of the transmission of H-polarized electromagnetic waves through a slot in a thick and perfectly conducting screen, formulated in terms of singular integral equations, combines the Nyström method with a specialized quadrature scheme based on variable transformation methods to handle kernel and geometric singularities.

In [22], Koshovy constructs correct asymptotical models of wave scattering from sparsely filled gratings of electrically narrow strips starting from log-singular and Cauchy-type integral equations. Nicholls [23] provides a high-order perturbative analysis of the Dirichlet–Neumann operator in a nonlinear Kerr medium via an interface-based method. The review paper by Tsitsas [24] presents an overview of integral-equation methods for all-dielectric isotropic grating, including boundary and volume integral equation methods, analytical regularization methods, the extended boundary condition method and methods employing auxiliary sources. Yin & Zhang [25] revisit and outline highly accurate boundary integral equation solvers for solving the acoustic, elastic and electromagnetic layered-medium scattering problems, especially the Windowed Green’s Function technique and the perfectly matched layer-boundary integral equation method.

## Data Availability

This article has no additional data.
